# The effect of respiratory gating strategy on left ventricular cardiac strains with DENSE

**DOI:** 10.1186/1532-429X-17-S1-Q107

**Published:** 2015-02-03

**Authors:** Sean M Hamlet, Kristin Andres, Gregory J Wehner, Jonathan D Suever, David K Powell, Xiaodong Zhong, Frederick H Epstein, Brandon K Fornwalt

**Affiliations:** 1 Electrical and Computer Engineering, University of Kentucky, Lexington, KY, USA; 2 MR R&D Department, Siemens Healthcare, Atlanta, KY, USA; 3 Pediatrics, University of Kentucky, Lexington, KY, USA; 4 Biomedical Engineering, University of Kentucky, Lexington, KY, USA; 5 Biomedical Engineering, University of Virginia, Charlottesville, VA, USA

## Background

**Figure 1:**
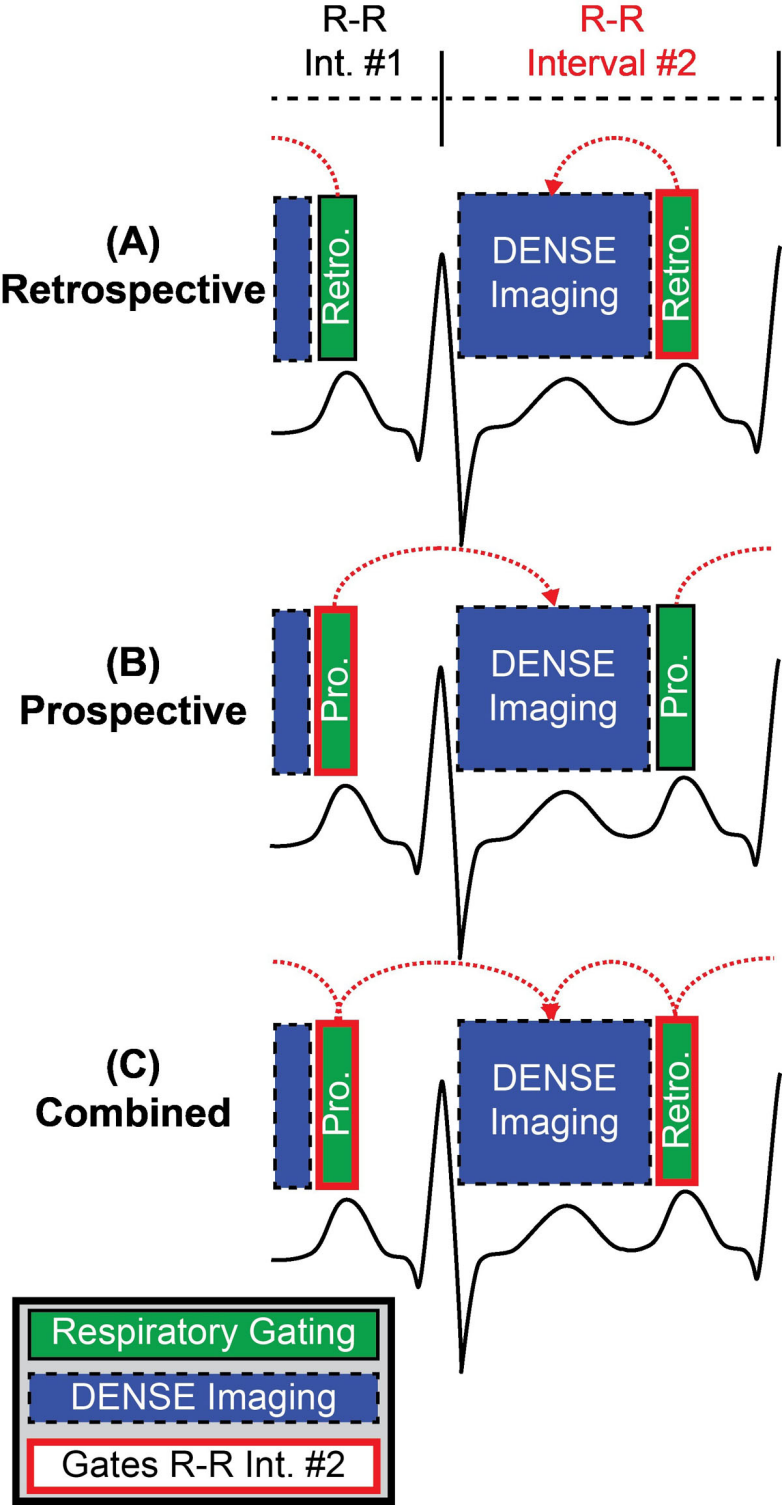
Different respiratory gating strategies to acquire DENSE image data for R-R interval #2. (A) Retrospective gating requires the diaphragm location to be within the acceptance window immediately following DENSE imaging in order to utilize DENSE images acquired during R-R interval #2. (B) Prospective gating requires the diaphragm location to be within the acceptance window before DENSE imaging (during R-R interval #1) in order to utilize DENSE images acquired during R-R interval #2. (C) Combined gating requires the diaphragm location both before and after DENSE imaging to be within the acceptance window in order to utilize DENSE images acquired during R-R interval #2.

Displacement Encoding with Stimulated Echoes (DENSE) directly measures tissue displacements and can be used to quantify cardiac mechanics. Multi-dimensional DENSE results in lengthy scans that require respiratory gating, acquiring data only while the diaphragm is within a pre-specified "acceptance window." Because it is not possible to perform respiratory gating during data acquisition, DENSE can employ the following respiratory gating strategies: 1) acquire data and keep it if the diaphragm is inside the window after acquisition (retrospective) 2) acquire data and keep it only if the diaphragm was inside the window right before data acquisition (prospective) or 3) a combination of retrospective and prospective where the diaphragm must be inside the window both before and after data acquisition (combined) (Fig 1). Combined respiratory gating is not used often because more data is discarded, resulting in longer scan times. It is possible, however, that with only retrospective or prospective respiratory gating, the diaphragm may not be within the acceptance window for the entirety of data acquisition (i.e. drifting into or out of the window), negatively affecting image quality. We hypothesized that the combined respiratory gating would result in significantly different estimates of left ventricular strains compared to either retrospective or prospective respiratory gating.

## Methods

2D Spiral cine DENSE of a mid-ventricular short-axis and 4-chamber long-axis image was performed on a 3T Siemens Tim Trio using the three strategies of respiratory gating: retrospective, prospective, and combined (Fig 1) in 10 healthy adults (Age: 26±9 years, 50% female) and 9 healthy children (Age 14±3 years, 44% female). Additional imaging parameters: 6 spiral interleaves, voxel size: 2.8x2.8x8 mm, TE/TR: 1.08/17, variable flip angle: 20°, 1 average, acceptance window: ±3mm. Respiratory gating efficiency and cardiac strains (radial, circumferential, and longitudinal) were determined for each subject and compared between combined respiratory gating and retrospective and prospective gating strategies using a student's t-test.

## Results

Using a retrospective or prospective gating strategy reduced scan time by an average of 41% compared to the combined approach (Table 1). However, these same strategies resulted in significantly different peak circumferential strains (p = 0.006 and p = 0.03, respectively) in children, but not adults.

**Table 1:** Bland-Altman statistics and t-test p-values for strains and respiratory gating efficiency for the various respiratory gating strategies (p < 0.05 indicates statistical significance and is denoted with a star).

	Adults		Children	
	Bias ± 95% Limits	p-value	Bias ± 95% Limits	p-value

Circumferential Strain (%)				

Retrospective vs. Combined	-0.8 ± 4.4	0.27	1.5 ± 2.1	0.006*

Prospective vs. Combined	-0.7 ± 4.3	0.33	1.2 ± 2.4	0.03*

Radial Strain (%)				

Retrospective vs. Combined	-1.9 ± 16.9	0.50	-0.3 ± 10.1	0.87

Prospective vs. Combined	2.1 ± 12.9	0.33	-1.7 ± 9.7	0.36

Longitudinal Strain (%)				

Retrospective vs. Combined	0.1 ± 3.3	0.81	-1.1 ± 4.9	0.25

Prospective vs. Combined	1.9 ± 4.2	0.02*	0.6 ± 3.3	0.37

Respiratory Gating Efficiency (%)				

Retrospective vs. Combined	22.8 ± 27.6	<0.001*	21.1 ± 16.9	<0.001*

Prospective vs. Combined	21.3 ± 28.4	0.001*	27.1 ± 11.0	<0.001*

## Conclusions

A retrospective or prospective respiratory gating strategy for DENSE imaging can reduce acquisition times by approximately 41%; however, a combined strategy should be utilized in children due to significant errors observed in peak circumferential strains measured using prospective or retrospective strategies. These differences were likely due to the fact that children have higher respiratory rates leading to a greater chance that retrospective and prospective gating strategies would have acquired DENSE data while the diaphragm was not within the acceptance window.

## Funding

This work was supported by a National Institutes of Health (NIH) Director's Early Independence Award (DP5 OD-012132); the University of Kentucky Cardiovascular Research Center, grant UL1RR033173 from the National Center for Research Resources (NCRR), funded by the Office of the Director, National Institutes of Health (NIH) and supported by the NIH Roadmap for Medical Research. The content is solely the responsibility of the authors and does not necessarily represent the official views of NIH.

